# Non-Contact Phenotypic Measurement and Body Mass Prediction of *Penaeus japonicus* Based on Skeletonization and Multi-View Comparison

**DOI:** 10.3390/s26154892

**Published:** 2026-08-03

**Authors:** Xuanyu Du, Junpeng Qu, Baoquan Yin

**Affiliations:** Yantai Institute of China Agricultural University, Yantai 264670, China; duxuanyu@cau.edu.cn (X.D.); 15355244316@163.com (J.Q.)

**Keywords:** *Penaeus japonicus*, non-contact measurement, skeletonization, computer vision, body mass prediction

## Abstract

To address systematic errors in prawn total length measurement caused by natural curvature and challenges in body mass prediction under random postures in aquaculture, this study proposes an automated phenotypic measurement and body mass prediction framework integrating skeleton-based nonlinear length estimation and multi-view feature analysis. Instance segmentation (YOLO11n-seg) obtains prawn head and abdomen masks. A skeleton-based procedure combining Zhang–Suen thinning, branch pruning, and graph-based path extraction obtains the projected body centerline for curved length measurement, while a curvature index quantifies body curvature. Body mass prediction models are built for side-view and top-view data, with SHAP analysis interpreting feature contributions. A unified random forest model using consistent morphological features enables body mass prediction across side-view and top-view samples. When evaluated against the independently acquired manual two-segment reference, the skeleton-based method achieved an MAE of 0.399 cm for severely curved prawns, representing reductions of 70.3%, 66.0%, and 21.9% relative to the straight-line method, MBR method, and Zhang–Suen baseline, respectively. On an independent test set, side-view and top-view models achieved mean absolute percentage errors of 5.73% and 5.82%, respectively. The unified RF model achieved an overall MAPE of 5.54%, and paired comparisons did not detect statistically significant differences from the corresponding viewpoint-specific models on either test subset. These results demonstrate the effectiveness of the proposed framework for non-contact prawn phenotyping under controlled imaging conditions.

## 1. Introduction

*Penaeus japonicus* (also referred to as *Marsupenaeus japonicus*) is taxonomically classified within the family Penaeidae and the genus *Penaeus* [1]. It is commonly referred to as “flower prawn” or “bamboo shrimp,” among other vernacular names, and is primarily distributed along the southeastern coastal regions of China, Japan, the Korean Peninsula, the eastern coast of Africa, and tropical waters of the Indo–West Pacific region [2]. Due to its desirable flesh quality, attractive appearance, and high nutritional value, as well as strong market demand and well-established aquaculture techniques, *Penaeus japonicus* has become an important marine aquaculture species with substantial economic value. In 2023, the mariculture area of *Penaeus japonicus* in China reached 22,000 hm^2^, with a total production of 45,970 t [3]. Under high-density aquaculture systems, precise feeding, growth assessment, and size grading rely on the rapid and accurate acquisition of phenotypic parameters (such as total length and body mass) [4]. Traditional manual measurement methods are associated with low efficiency and substantial subjective errors [5]. Therefore, it is of great importance to develop a non-contact and automated system for prawn phenotypic measurement and body mass prediction.

In recent years, computer vision technologies based on deep learning have been widely applied in aquaculture [6,7,8]. In image segmentation tasks, fully convolutional networks (such as U-Net), with encoder–decoder architectures and skip connections, have demonstrated strong edge-preserving capabilities in biomedical image segmentation and have subsequently been widely adopted for fish and shrimp contour extraction [9]. At the level of object detection and instance segmentation, single-stage models represented by the YOLO series are widely favored due to their high inference speed and ease of deployment. For example, YOLOv8-seg has been applied to prawn instance segmentation and counting [10], and Correia et al. developed an automated monitoring system for prawn total length and body mass based on YOLOv8-seg [11]. Zhang Shixuan et al. integrated HRNet-based keypoint detection with Mask R-CNN to construct a high-throughput phenotypic measurement system for *Litopenaeus vannamei*, achieving high accuracy in both total length measurement and body mass prediction [12]. However, existing vision-based methods for total length extraction predominantly rely on the minimum bounding rectangle or straight-line distances between key points [13,14]. For prawns with flexible bodies that commonly exhibit varying degrees of curvature under natural conditions or on conveyor belts, such approaches introduce systematic measurement errors that are positively correlated with curvature levels [15]. Although Lai et al. attempted to measure prawn total length in practical aquaculture scenarios by combining threshold segmentation with the minimum bounding rectangle, the accuracy remains limited by body overlap and postural curvature effects [14].

To overcome the limitations imposed by rigid length-measurement assumptions, morphological skeletonization provides a feasible solution. Luo et al. employed binary image thinning to extract the principal skeleton of prawns for total length calculation [16]. Zhou et al. integrated Mask R-CNN segmentation with skeletonization post-processing for automated prawn size measurement [17], whereas Waqar et al. combined instance segmentation with centerline prediction in a dual-segmentation framework [15]. For overlapping fish images, Zhao et al. combined convex–concave contour segmentation with head–tail matching to extract and optimize individual centerlines [18]. These studies demonstrate the applicability of skeleton- and centerline-based approaches to different aquaculture imaging tasks. In the present study, the straight-line and MBR methods were included as rigid comparison methods, whereas an unpruned Zhang–Suen implementation provided a direct skeletonization baseline. Comparisons across curvature groups were conducted to assess skeleton-path measurement against rigid methods and to evaluate the combined effects of regional mask extraction and branch pruning against the Zhang–Suen baseline.

For body mass prediction, the classical allometric power-law regression model (PLR), $W=aL^{b}$, has been widely used in aquaculture to describe the allometric relationship between total length and body mass in prawns [19]. However, this univariate power-law model relies exclusively on total length and neglects the additional explanatory value provided by multidimensional morphological features, such as body height, body width, and projected area. In recent years, machine learning methods, including multiple linear regression (MLR), support vector regression (SVR), and random forest (RF), have been increasingly adopted to improve body mass prediction accuracy by integrating multidimensional morphological features [20,21,22]. Chirdchoo et al. extracted features including area, perimeter, total length, body width, and posture from prawn images and employed artificial neural networks for body mass prediction, demonstrating that the incorporation of multidimensional morphological features effectively improved estimation accuracy [21]. Setiawan et al. estimated prawn body mass using underwater image analysis and machine learning techniques based on morphometric features; however, the feature set was limited to basic descriptors, such as area and perimeter [7]. Nevertheless, most existing studies have been conducted using a single imaging perspective. Consequently, the relative merits of side-view imaging, which provides body height and lateral projected area, and top-view imaging, which provides body width and projected area, have not been systematically compared for body mass prediction. Furthermore, a general solution capable of handling randomly oriented prawns using a single model without prior posture classification has not yet been explored.

To address these limitations, this study proposes a prawn phenotypic measurement and body mass prediction method that integrates skeleton-based nonlinear length measurement with multi-view comparative analysis. First, we implemented and evaluated a skeleton-based length measurement method combining Zhang–Suen thinning, branch pruning, and graph-based shortest-path search against the straight-line method, MBR method, and Zhang–Suen baseline across different curvature groups. Next, we established separate body mass prediction models for side-view and top-view data and used SHAP to interpret the relative contributions of the predefined morphological features. Finally, we developed a unified model to evaluate mixed-posture prediction using a single model.

## 2. Materials and Methods

**Figure 1:**
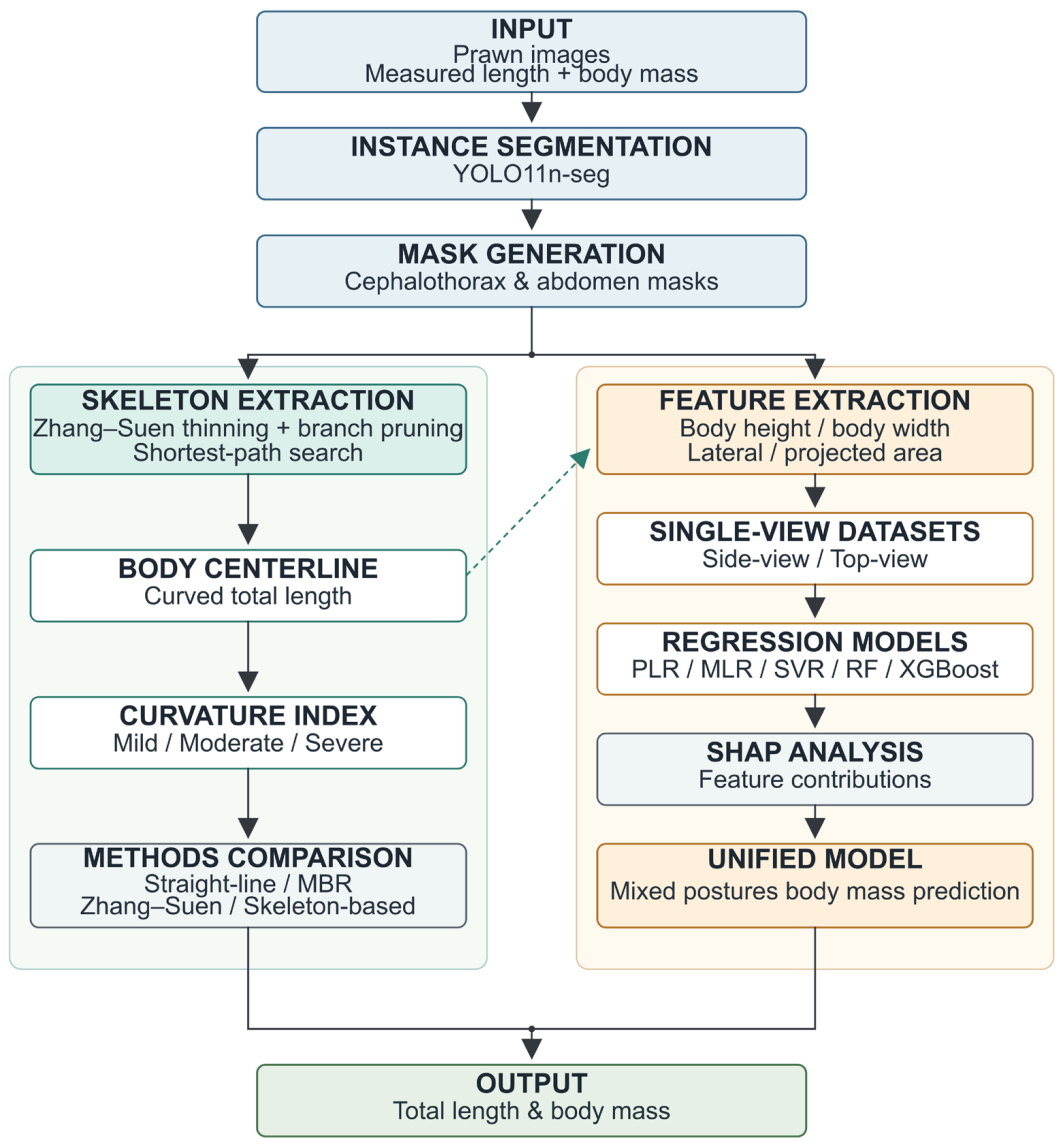
Overall workflow of the proposed framework for prawn total length measurement and body mass prediction.

The overall workflow of the proposed framework is illustrated in Figure 1. Following instance segmentation and regional mask generation, skeleton-based total length measurement and multi-view body mass prediction were performed through two interconnected branches.

### 2.1. Data Acquisition

The experimental samples used in this study were *Penaeus japonicus*, collected from the Shun Hexing Marine Hatchery in Penglai District, Yantai City, Shandong Province, China. After screening, 347 valid live specimens were obtained, with total lengths ranging from 8.0 to 19.9 cm and body masses ranging from 8.6 to 36.0 g. Image acquisition was performed under controlled laboratory conditions. A Redmi K60 smartphone (Xiaomi Communications Co., Ltd., Beijing, China; image resolution: 3472 × 4624 pixels) was mounted approximately 40 cm above the experimental platform and used to capture vertically downward images. A standard stainless-steel graduated ruler was placed within the field of view as a physical scale reference.

To reduce background noise, a white matte background paper was placed on the imaging surface. Each prawn was placed naturally on the background in an arbitrary posture. The natural orientation of the prawn on the platform determined its imaging view: individuals with the lateral side facing upward were classified as the side-view group, whereas those with the dorsal side facing upward were classified as the top-view group. Only one image was acquired per individual. After imaging, each specimen was assigned to either the side-view or top-view group according to its actual orientation in the image. A total of 347 original images were collected.

**Figure 2:**
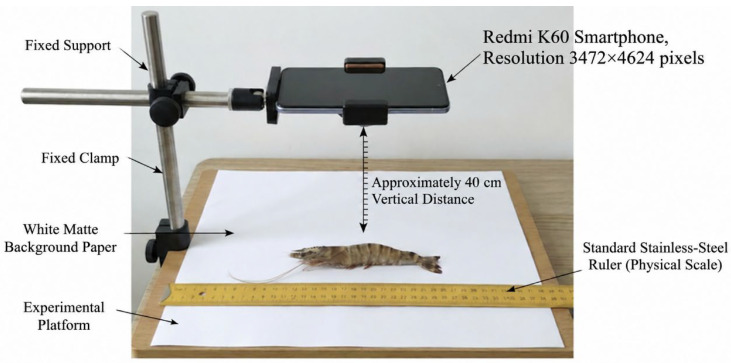
Schematic diagram of the image acquisition system.

Simultaneously, a vernier caliper (accuracy: 0.1 mm) was used to obtain the reference total length while each prawn remained in its natural posture. The measurement was divided at the cephalothorax–abdomen junction: the distance from the rostrum tip to the junction and that from the junction to the distal end of the tail fan were measured separately, and their sum was recorded as the reference total length. The reference measurement was obtained independently of the image-processing results. All specimens were measured by the same operator using the same anatomical landmarks and two-segment procedure. Body mass was measured using a digital electronic balance (accuracy: 0.1 g), and all measurements were linked to the corresponding image identifiers. The imaging setup is shown in Figure 2.

### 2.2. Instance Segmentation Model

The YOLO11n-seg instance segmentation model was implemented using Ultralytics 8.3.163 and employed as the baseline architecture. The architecture incorporates the C3k2 module and the C2PSA attention mechanism, thereby preserving lightweight characteristics while achieving strong feature extraction capability [23]. During training, the input image size was set to 1024 × 1024 pixels with a batch size of 8. The stochastic gradient descent (SGD) optimizer was used with an initial learning rate of 0.003 and a weight decay coefficient of 0.002. The model was trained for 100 epochs.

To enhance sample diversity and improve training robustness, an online data augmentation strategy was applied during training, including random adjustments of hue, saturation, and brightness, as well as small-range translation and scaling. To improve mask refinement quality, the loss function weights were biased toward mask accuracy during training. During the validation stage, the intersection-over-union (IoU) threshold was set to 0.7 to balance detection accuracy and computational efficiency.

### 2.3. Dataset Construction

Before data augmentation, the 347 original images were divided into training, validation, and test sets in a ratio of 7:2:1. To increase sample diversity and improve model generalization, offline augmentation was subsequently performed independently within each subset, ensuring that augmented images derived from the same original image remained in the same subset. Data augmentation artificially expands the training dataset and increases training diversity, thereby improving model performance across multiple tasks [24]. The augmentation scheme adopted in this study included random brightness and contrast adjustments (±15%), geometric transformations, and noise injection. A total of 1388 *Penaeus japonicus* images were ultimately obtained.

All images were annotated using the LabelMe 5.8.1 with polygon annotations for two categories: head and abdomen. The rationale for dividing the prawn body into head and abdomen is that cephalothorax length and abdomen length can be extracted separately using a skeletonization algorithm, and their sum is used as the total length. Compared with directly extracting total length from the full prawn mask, segmented extraction enables more precise localization of the boundary between the cephalothorax and abdominal segments, thereby providing a more accurate morphological basis for calculating the curvature index (CI).

**Figure 3:**
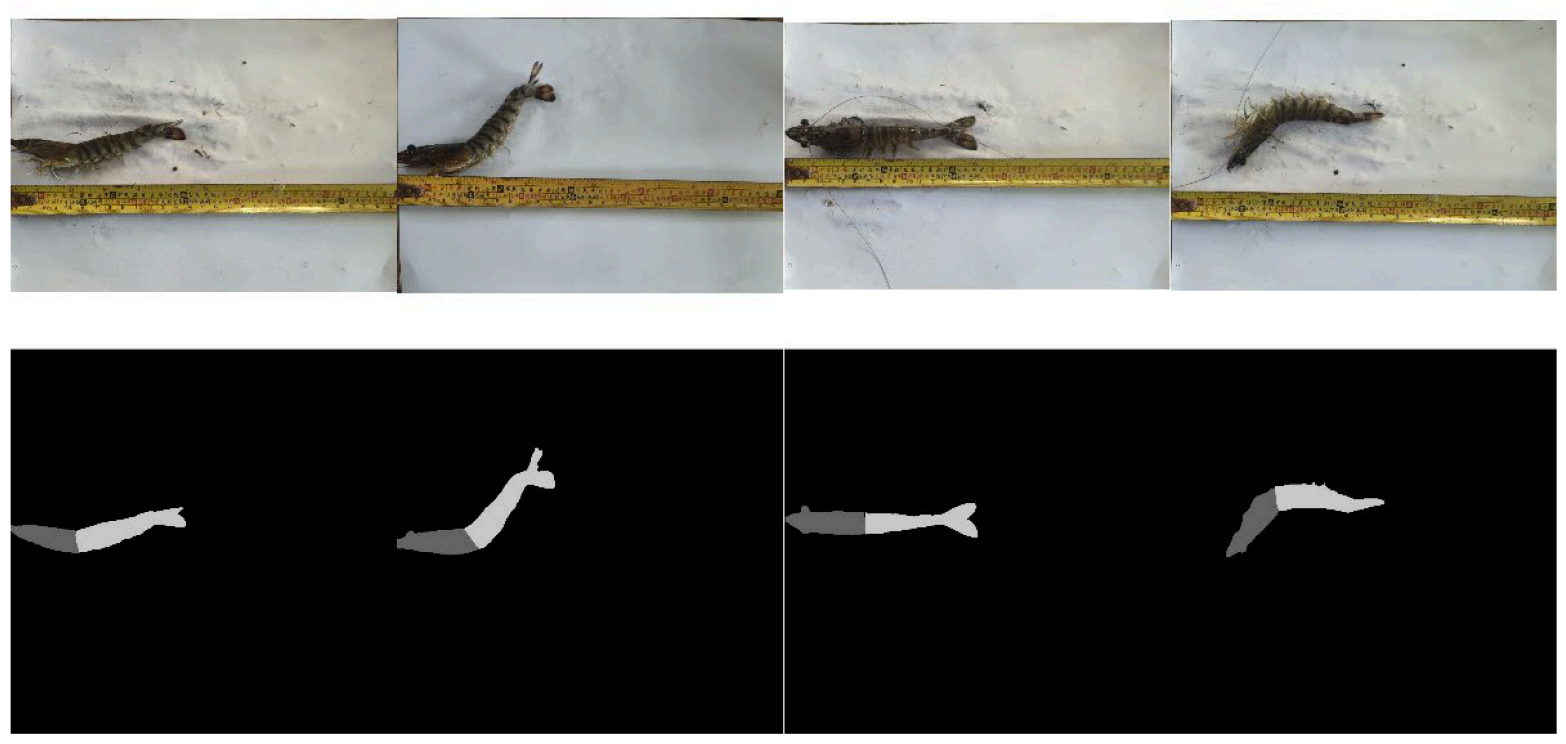
Example of mask annotation in the dataset. The dark-gray and light-gray regions represent the head (pixel value 100) and abdomen (pixel value 200) masks, respectively.

After annotation, pixel-level semantic masks were generated. In these masks, background pixels were assigned a value of 0, the head region a value of 100, and the abdomen region a value of 200, allowing the prawn head and abdomen to be distinguished via different grayscale labels. Representative examples of annotated masks are shown in Figure 3.

### 2.4. Prawn Total Length Measurement Method Based on Skeletonization

#### 2.4.1. Skeleton Extraction and Curvature Index Calculation

To address the systematic errors introduced by traditional rigid length-measurement methods due to the natural curvature of prawn bodies, this study implemented a skeleton-based length measurement method integrating Zhang–Suen thinning, branch pruning, and graph-based shortest-path search. By leveraging the separate head and abdomen annotations described in Section 2.3, the cephalothorax length and abdomen length are extracted independently and then summed to obtain the total length, thereby reducing localization errors at the cephalothorax–abdominal junction that may arise when using a single skeleton arc length under curved postures.

For the obtained binary prawn masks, the Zhang–Suen thinning algorithm [25] was applied to extract a one-pixel-wide skeleton centerline. Skeletonization is sensitive to small boundary details and may generate redundant branches [26]. Since appendages such as the rostrum, pereiopods, and tail fan may introduce redundant short branches in the skeleton and interfere with accurate centerline extraction, a branch-pruning procedure was applied to reduce their influence. All endpoints and branch points in the skeleton were detected, and short branches with lengths below a predefined threshold (15 pixels) were treated as appendage noise and removed. This process was iteratively repeated until only a smooth centerline of the prawn body remained.

For each pruned regional skeleton, an undirected weighted graph was constructed as follows:

(1) $$G=\left(V,E\right)$$

where G is an undirected weighted graph constructed from the pixels of a regional skeleton, V denotes the set of skeleton pixels, and E denotes the edges connecting adjacent pixels within an 8-neighborhood. The edge weights were defined as the Euclidean distances between adjacent pixels. For each regional graph, all degree-one nodes were identified as candidate endpoints, and Dijkstra’s algorithm [27] was used to calculate the shortest-path length between each endpoint pair. The endpoint pair yielding the maximum shortest-path length was selected to define the principal centerline of the corresponding region. The cephalothorax and abdomen centerline lengths were then summed to obtain L_curve. The Euclidean distance between the outer endpoints of the two regional centerlines, corresponding to the rostrum tip and the distal end of the tail fan, was recorded as L_straight. Both measurements represent two-dimensional projected quantities in the image plane.

To quantitatively characterize the curvature of prawn bodies, the curvature index (CI) was adopted as a morphological descriptor. CI is defined as the ratio of skeleton arc length to straight-line distance:

(2) $$CI=\frac{L\_curve}{L\_straight}$$

where L_curve denotes the summed arc length of the cephalothorax and abdomen centerlines, and L_straight denotes the Euclidean distance between the rostrum tip and the tail fan endpoint.

**Figure 4:**
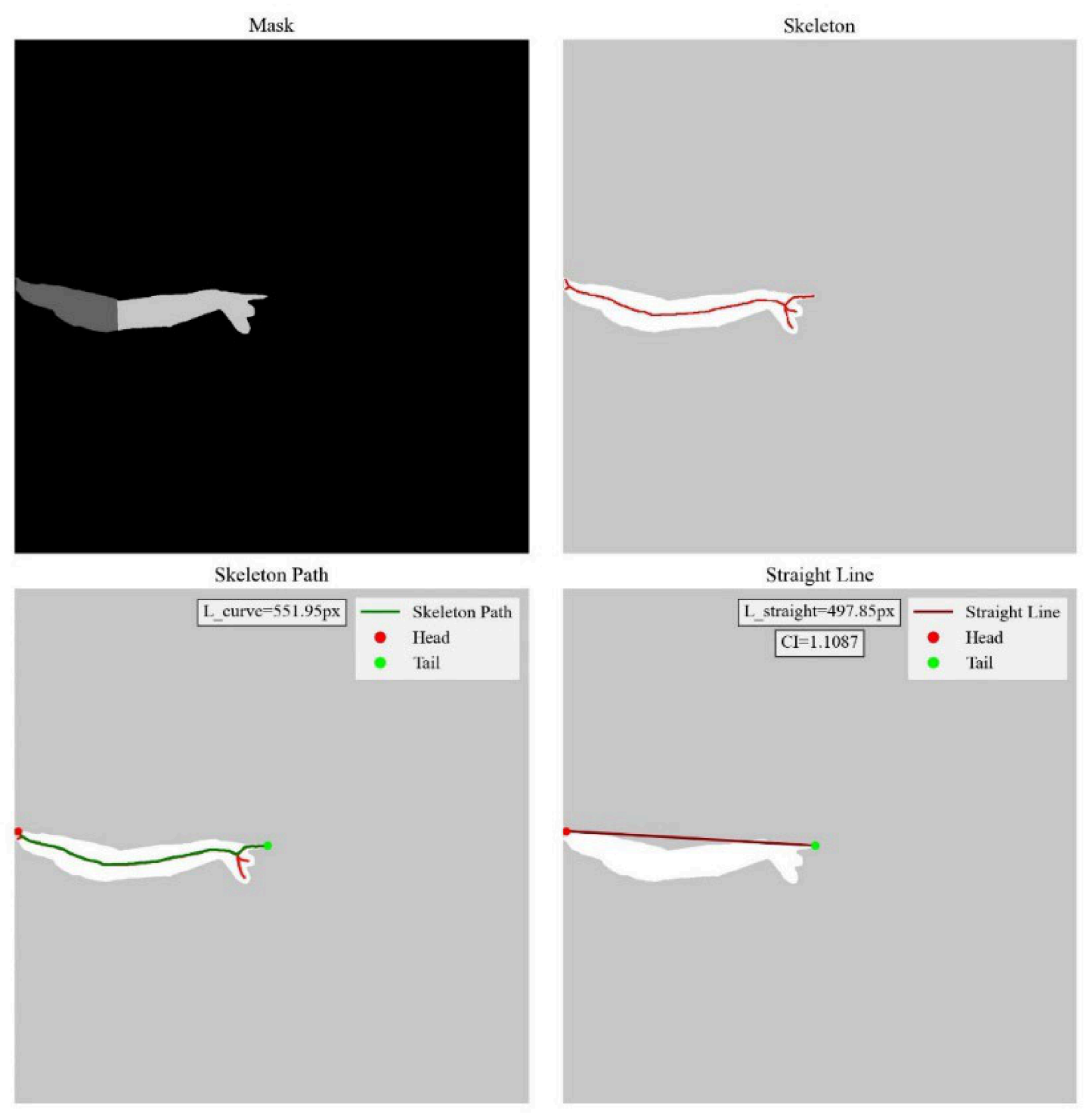
Schematic diagram of curvature index calculation.

Figure 4 illustrates the calculation of skeleton arc length and straight-line distance for a representative prawn sample. The green curve represents the skeleton-based arc length L_curve, while the red line represents the Euclidean distance L_straight between the two endpoints. The ratio of these two values corresponds to the CI. A CI value closer to 1.0 indicates a straighter body posture, whereas a higher CI value indicates a greater degree of curvature. Based on the observed distribution of CI values, thresholds of 1.08 and 1.15 were established prior to error analysis to provide consistent stratification of curvature severity and clearer differentiation across the mild-to-moderate curvature range. Samples were accordingly classified as mildly curved (CI ≤ 1.08), moderately curved (1.08 < CI ≤ 1.15), and severely curved (CI > 1.15).

#### 2.4.2. Comparison of Four Length Measurement Methods

**Figure 5:**
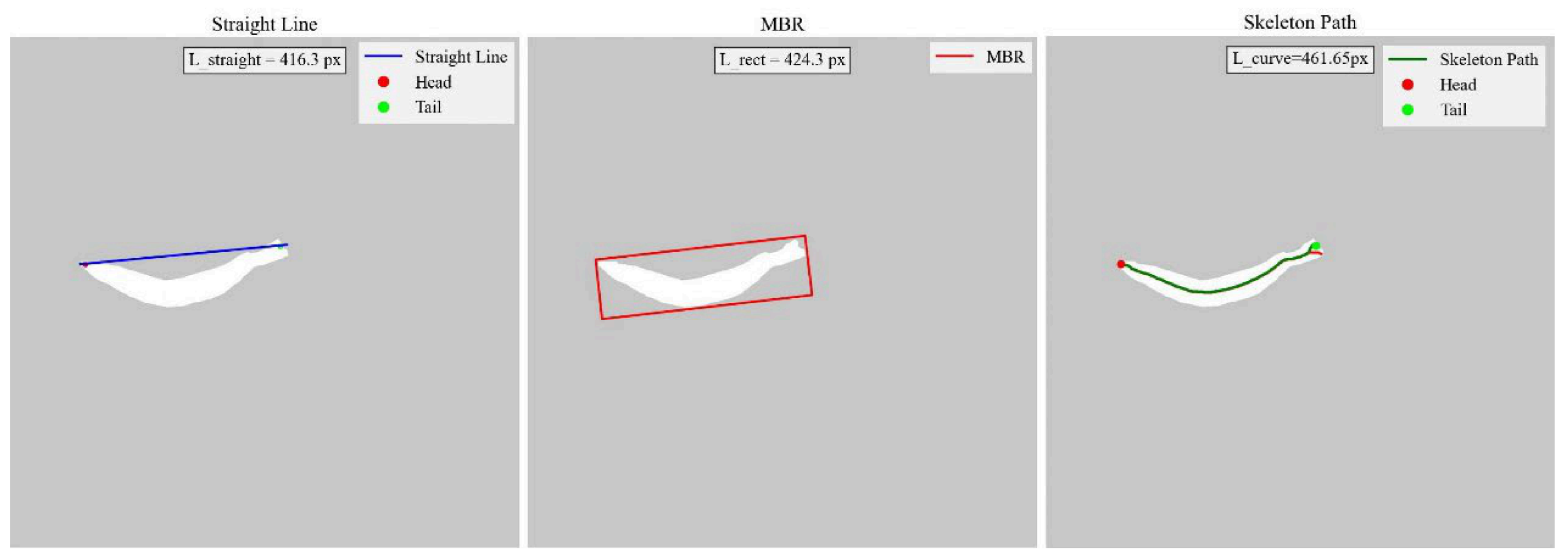
Schematic illustration of straight-line, MBR, and skeleton-path measurements.

To systematically evaluate the accuracy of the skeleton-based method, two conventional rigid length measurement methods and a Zhang–Suen baseline were included for comparison. Figure 5 schematically illustrates the straight-line, MBR, and skeleton-path measurement principles. The Zhang–Suen baseline and skeleton-based method both employ skeleton-path measurement but differ in mask extraction and branch pruning, as described below.

Straight-line method: The manually annotated rostrum tip and tail fan endpoint are used as reference endpoints. Taking the line connecting these two points as the primary projection axis, the difference between the maximum and minimum projections of all prawn foreground pixels onto this axis is computed as the measured length. This method approximates total length via projection span, simulating manual ruler-based measurement aligned with the head-to-tail direction.

Minimum bounding rectangle (MBR) method: The minimum-area rotated rectangle enclosing all foreground pixels of the prawn is computed, and the length of its longer side is used as the measurement result [5]. This method does not rely on manually annotated keypoints, and the orientation and dimensions of the rectangle are entirely determined by the spatial distribution of foreground pixels. It is one of the most widely used rigid measurement approaches in industrial vision systems and has been extensively applied to automated prawn total length measurement in aquaculture environments [8,13,14].

Zhang–Suen baseline: The full prawn mask was skeletonized using Zhang–Suen thinning without the subsequent branch-pruning operation. All degree-one nodes were identified as candidate endpoints, and the endpoint pair with the maximum shortest-path length was selected. The length of the corresponding skeleton path was taken as the total length.

Skeleton-based method: This refers to the method described in Section 2.4.1, in which the principal shortest paths are extracted separately from the pruned cephalothorax and abdomen skeletons, and their lengths are summed to obtain total length.

The four length estimates were initially expressed in pixels. For each image, the endpoints of a 1 cm interval on the reference ruler were manually identified, and its pixel length was used to calculate an image-specific conversion coefficient (cm/px). This coefficient was then applied to convert all four estimates into centimeters for subsequent accuracy evaluation.

### 2.5. Comparative Analysis of Body Mass Prediction Between Side-View and Top-View Perspectives

#### 2.5.1. Feature Definition of Single-View Datasets

To evaluate the influence of different imaging perspectives on body mass prediction accuracy, we constructed two independent datasets comprising side-view and top-view images. Both datasets were acquired using the vertical downward imaging setup described in Section 2.1. Under the side-view condition, the maximum transverse extent perpendicular to the skeleton centerline was defined as body height, while the segmentation mask area represented the lateral projected area. Under the top-view condition, the corresponding transverse extent was defined as body width, while the mask area represented projected area.

All morphological features were computed based on the binary masks generated in Section 2.2 and the skeleton centerline extracted in Section 2.4.1. Specifically, total length was obtained using the skeleton-based measurement method described in Section 2.4.1, defined as the sum of cephalothorax length and abdomen length. The cephalothorax length and abdomen length were computed as the arc lengths of the skeleton centerline within the head region (mask value 100) and abdomen region (mask value 200), respectively. Body height was derived from side-view masks and defined as the maximum transverse extent perpendicular to the skeleton centerline. Body width was derived from top-view masks and defined in the same manner. Lateral projected area and projected area were calculated as the total number of foreground pixels in the side-view and top-view masks, respectively, and converted into physical units using the scale coefficient.

**Figure 6:**
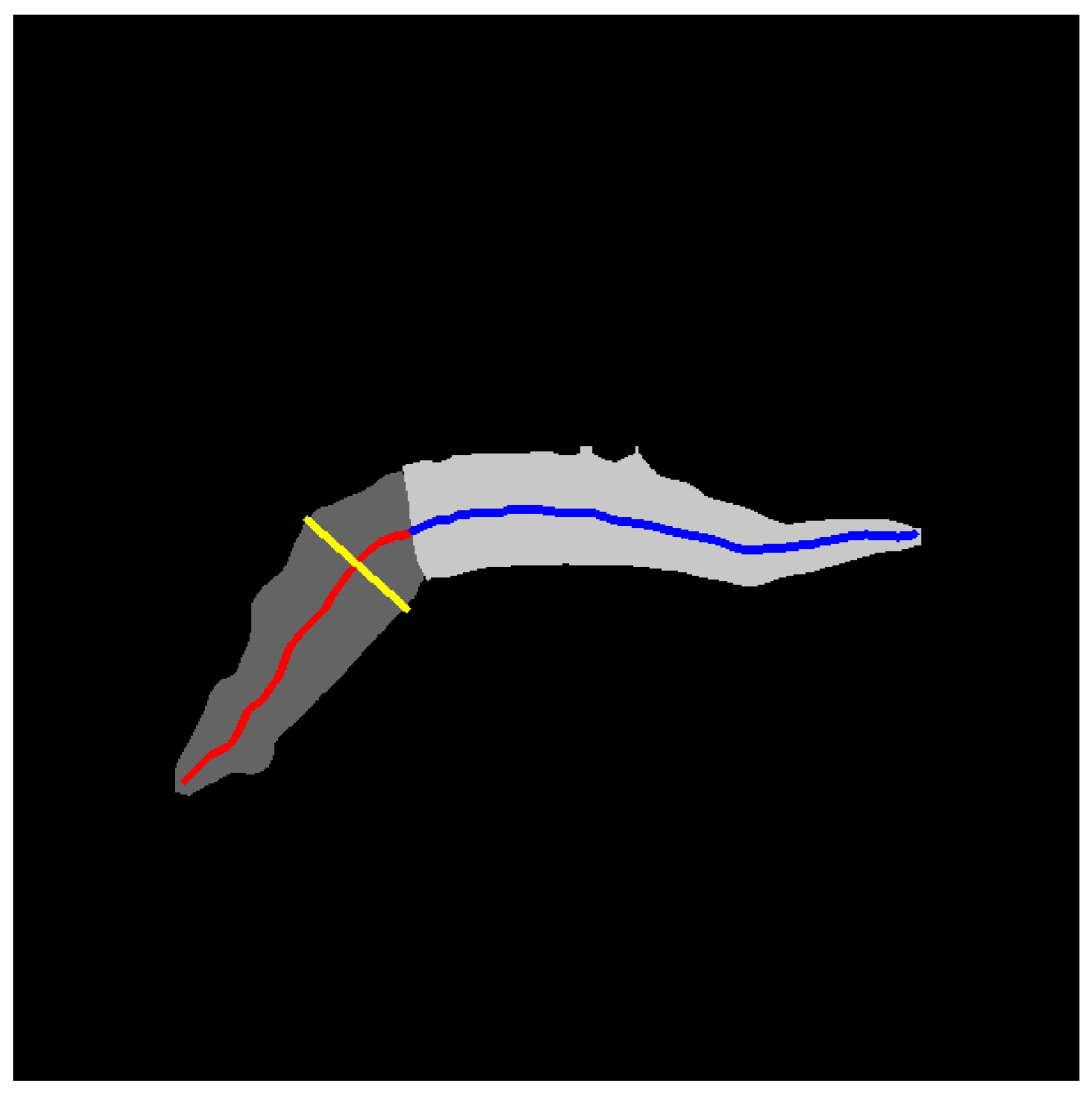
Schematic diagram of morphological feature extraction. Note: The red arc represents cephalothorax length, the blue arc represents abdomen length, and their sum constitutes total length; the yellow line segment represents the maximum transverse extent (body height in side view, body width in top view); the semi-transparent region indicates the calculation area for lateral projected area and projected area.

All length-related parameters were converted from pixel units to physical units (cm) using the scale ruler in the images, while area-related parameters were converted into cm^2^. The extraction of the above morphological features is illustrated in Figure 6.

The feature set for the side-view dataset is defined as [total length, cephalothorax length, abdomen length, body height, lateral projected area], whereas the feature set for the top-view dataset is defined as [total length, cephalothorax length, abdomen length, body width, projected area]. The target variable for both datasets is manually measured body mass (g).

#### 2.5.2. Regression Models

To systematically compare body mass prediction performance under the two imaging perspectives, five representative regression algorithms were selected, including PLR, MLR, SVR, RF, and XGBoost.

Power-law regression (PLR) is a classical model describing the length–weight relationship in aquaculture biology, expressed as $W=aL^{b}$. For parameter estimation, the nonlinear model was linearized via logarithmic transformation: $\ln\left(W\right)=\ln\left(a\right)+b\cdot \ln\left(L\right)$. The transformed model was fitted using the least squares method, and predictions were converted back to body mass using the exponential function. This model uses only total length (L) as a single feature and serves as a baseline for comparison with multivariate models [19].

Multiple linear regression (MLR) fits the linear relationship between body mass and each morphological feature using the least squares method and provides an interpretable measure of each feature’s linear contribution to body mass [28].

Support vector regression (SVR) employs a radial basis function kernel to map features into a high-dimensional space and uses an ε-insensitive loss function for robust regression. It exhibits strong nonlinear fitting capability in small-sample scenarios [29]. In this study, SVR was implemented using scikit-learn 1.8.0 with C = 1.0, γ = “scale”, and ε = 0.1; the remaining parameters retained their default values.

Random forest (RF) is an ensemble learning method based on bagging. It constructs multiple decision trees using bootstrap sampling from the original dataset, and the final prediction is obtained by averaging the outputs of all trees [30]. In this study, RF was implemented using scikit-learn 1.8.0 with n_estimators = 200, max_depth = None, min_samples_split = 2, min_samples_leaf = 1, max_features = 1.0, bootstrap = True, and random_state = 42; the remaining parameters retained their default values.

Extreme gradient boosting (XGBoost) is a gradient-boosted tree algorithm that constructs an additive ensemble of decision trees and incorporates regularization to control model complexity [31]. In this study, XGBoost was implemented using XGBoost 3.2.0 with n_estimators = 100, max_depth = 6, learning_rate = 0.3, subsample = 1.0, colsample_bytree = 1.0, reg_alpha = 0.0, reg_lambda = 1.0, objective = “reg:squarederror”, and random_state = 42; the remaining parameters retained their default values.

PLR, MLR, SVR, and RF were implemented using scikit-learn 1.8.0, whereas XGBoost was implemented using XGBoost 3.2.0. Default hyperparameters were adopted unless otherwise specified, and no further hyperparameter tuning was performed.

For MLR and SVR, feature standardization was applied prior to training, whereas RF and XGBoost were trained directly on raw features. It should be noted that a perfect linear dependency exists among total length, cephalothorax length, and abdomen length (total length = cephalothorax length + abdomen length). For the MLR model, such multicollinearity leads to instability in coefficient estimation; therefore, only overall predictive performance is reported, without interpreting individual coefficients. For SVR, RF, and XGBoost, the complete feature set was retained because the primary objective was predictive comparison rather than coefficient interpretation. Total length was retained to maintain consistency with the PLR baseline.

#### 2.5.3. Model Evaluation, Statistical Analysis and SHAP Interpretation

For the instance segmentation task, several commonly used metrics were employed to quantitatively evaluate mask quality, including the Dice coefficient, the 95% Hausdorff distance (HD95), and the average surface distance (ASD).

(3) $$Dice=\frac{2\left|P⋂G\right|}{\left|P\right|+\left|G\right|}$$

(4) $$HD95={percentile}_{95}\left\{d\left(P,G\right)\right\}$$

(5) ASD=1P+G∑p∈P,g∈G dp,g

where *p* and *G* denote the predicted segmentation mask and the ground-truth mask, respectively.

All models were evaluated using repeated five-fold cross-validation and an independent test set. Approximately 10% of the samples from each dataset were held out before model evaluation and were not used for training, parameter specification, or cross-validation. The remaining samples were evaluated using five-fold cross-validation repeated 10 times with different random partitions, and the mean and standard deviation across the 50 validation folds were used to assess model stability. Given the size of the held-out subsets, independent testing was treated as an initial out-of-sample assessment, while repeated cross-validation and bootstrap confidence intervals were used to characterize performance stability and uncertainty.

In this study, four metrics were used to evaluate regression performance: coefficient of determination ($R^{2}$), mean absolute error (MAE), root mean square error (RMSE), and mean absolute percentage error (MAPE). The definitions of these metrics are given as follows:

(6) $$R^{2}=1-\frac{\sum_{i=1}^{n}{\left(y_{i}-{\hat{y}}_{i}\right)}^{2}}{\sum_{i=1}^{n}{\left(y_{i}-\overset{ˉ}{y}\right)}^{2}}$$

(7) $$\mathrm{MAE}=\frac{1}{n}\sum_{i=1}^{n}|y_{i}-{\hat{y}}_{i}|$$

(8) $$\mathrm{RMSE}=\sqrt{\frac{1}{n}\sum_{i=1}^{n}{\left(y_{i}-{\hat{y}}_{i}\right)}^{2}}$$

(9) $$\mathrm{MAPE}=\frac{1}{n}\sum_{i=1}^{n}|\frac{y_{i}-{\hat{y}}_{i}}{y_{i}}|\times 100\%$$

where $y_{i}$ denotes the measured body mass of the i-th sample, ${\hat{y}}_{i}$ denotes the predicted body mass, $\overset{ˉ}{y}$ denotes the mean of measured body mass values, and n denotes the total number of samples in the evaluated dataset or validation fold.

The uncertainty associated with the independent-test metrics was quantified using nonparametric bootstrap resampling. A total of 2000 bootstrap samples were generated by resampling the paired measured and predicted values with replacement. Percentile-based 95% confidence intervals were subsequently calculated for MAE, RMSE, and MAPE.

To compare the predictive performance of the regression models, the sample-wise absolute percentage errors obtained from the same independent test specimens were treated as paired observations. The RF model was compared separately with the PLR, MLR, SVR, and XGBoost models using two-sided paired Wilcoxon signed-rank tests. The resulting *p*-values were adjusted separately within each imaging view using the Holm method. An adjusted *p*-value below 0.05 was considered statistically significant.

SHAP (SHapley Additive exPlanations) was employed for post-hoc interpretation of the trained RF model and comparison of the relative contributions of the predefined morphological features. SHAP is based on Shapley values from game theory and decomposes each prediction into the sum of marginal contributions of individual features, thereby quantifying both the magnitude and direction of each feature’s influence on the model output [32]. Because total length equals the sum of cephalothorax length and abdomen length, the SHAP values were interpreted as model-specific attributions among correlated predictors rather than as independent morphological effects.

### 2.6. Unified Body Mass Prediction Model for Mixed Postures

In practical aquaculture sorting systems, prawns transported on a conveyor may present either side-view or top-view orientations. A posture-specific strategy would require prior viewpoint identification and subsequent model selection, thereby increasing deployment complexity. To address this issue, a unified body mass prediction model for mixed postures was developed. By unifying feature definitions across viewpoints, the side-view and top-view datasets were merged to train a single model capable of handling multiple typical postures simultaneously.

The two independent datasets described in Section 2.5.1 (side-view and top-view) were merged, and feature definitions were unified. Body height in the side view and body width in the top view were collectively defined as the maximum transverse dimension, whereas lateral projected area in the side view and projected area in the top view were both defined as projected area. The resulting mixed-posture dataset was represented by the feature set [total length, cephalothorax length, abdomen length, maximum transverse dimension, projected area], and the target variable was manually measured body mass (g). Each sample was additionally assigned a viewpoint label (side-view/top-view) to enable subsequent evaluation of predictive performance across viewpoints.

Using the mixed-posture dataset as input, an RF model with the same hyperparameter configuration as described in Section 2.5.2 was trained. An evaluation strategy consistent with Section 2.5.3 was adopted, in which an independent test set was first held out, followed by five-fold cross-validation repeated 10 times on the remaining data. The independent test set was further split into side-view and top-view subsets according to viewpoint labels, and MAPE was computed separately for each subset to assess the predictive performance of the unified model for both posture types.

To compare the unified and viewpoint-specific models on identical test samples, sample-level absolute percentage errors were analyzed separately for the side-view and top-view subsets using two-sided paired Wilcoxon signed-rank tests. *p*-values were adjusted using the Holm method, and 95% confidence intervals for the mean paired MAPE difference were estimated using 2000 paired bootstrap resamples. To assess the influence of training sample size, a sample-size-matched ablation experiment was further conducted. The pooled training set was stratified by viewpoint and sampled without replacement to match the corresponding viewpoint-specific training size (*n* = 153 for side-view and *n* = 100 for top-view). This procedure was repeated 200 times, and each refitted model was evaluated on the corresponding fixed test subset.

## 3. Results and Analysis

### 3.1. Experimental Configuration

This study was conducted on a Windows 11 (Microsoft Corporation, Redmond, WA, USA) operating system equipped with an AMD Ryzen 9 7940H processor (Advanced Micro Devices, Inc., Santa Clara, CA, USA) and an NVIDIA GeForce RTX 4060 Laptop GPU. The deep learning framework used was PyTorch 2.5.0, the development environment was PyCharm 2025.1.1.1, and the programming language was Python 3.11.

### 3.2. Segmentation Model Performance

Table 1 summarizes the performance of the YOLO11n-seg instance segmentation model on the test set. The Dice coefficients for the head and abdomen were 0.931 and 0.950, respectively. The ASD values were 3.212 and 1.935 pixels, and the HD95 values were 10.755 and 6.996 pixels, respectively. The mAP@50:95 was 0.840.

The relatively low HD95 values indicate that large boundary deviations were limited for both categories, whereas ASD values below 5 pixels indicate close average agreement between the predicted and reference mask boundaries.

These results demonstrate that the YOLO11n-seg model provides high-quality and finely detailed prawn masks for subsequent skeleton extraction and morphological measurement, thereby satisfying the segmentation accuracy requirements of this study.

### 3.3. Comparison of Total Length Measurement Errors Across Different Curvature Groups

Measurement errors for all four methods were calculated relative to the independently acquired manual two-segment reference described in Section 2.1. According to the CI thresholds defined in Section 2.4.1, samples were divided into three groups. Forty-seven samples were excluded because the CI could not be reliably computed due to incomplete mask boundaries or abnormal skeleton branching. Ultimately, 300 valid samples were retained for comparison of the four length measurement methods, including 146 mildly curved, 114 moderately curved, and 40 severely curved samples.

**Figure 7:**
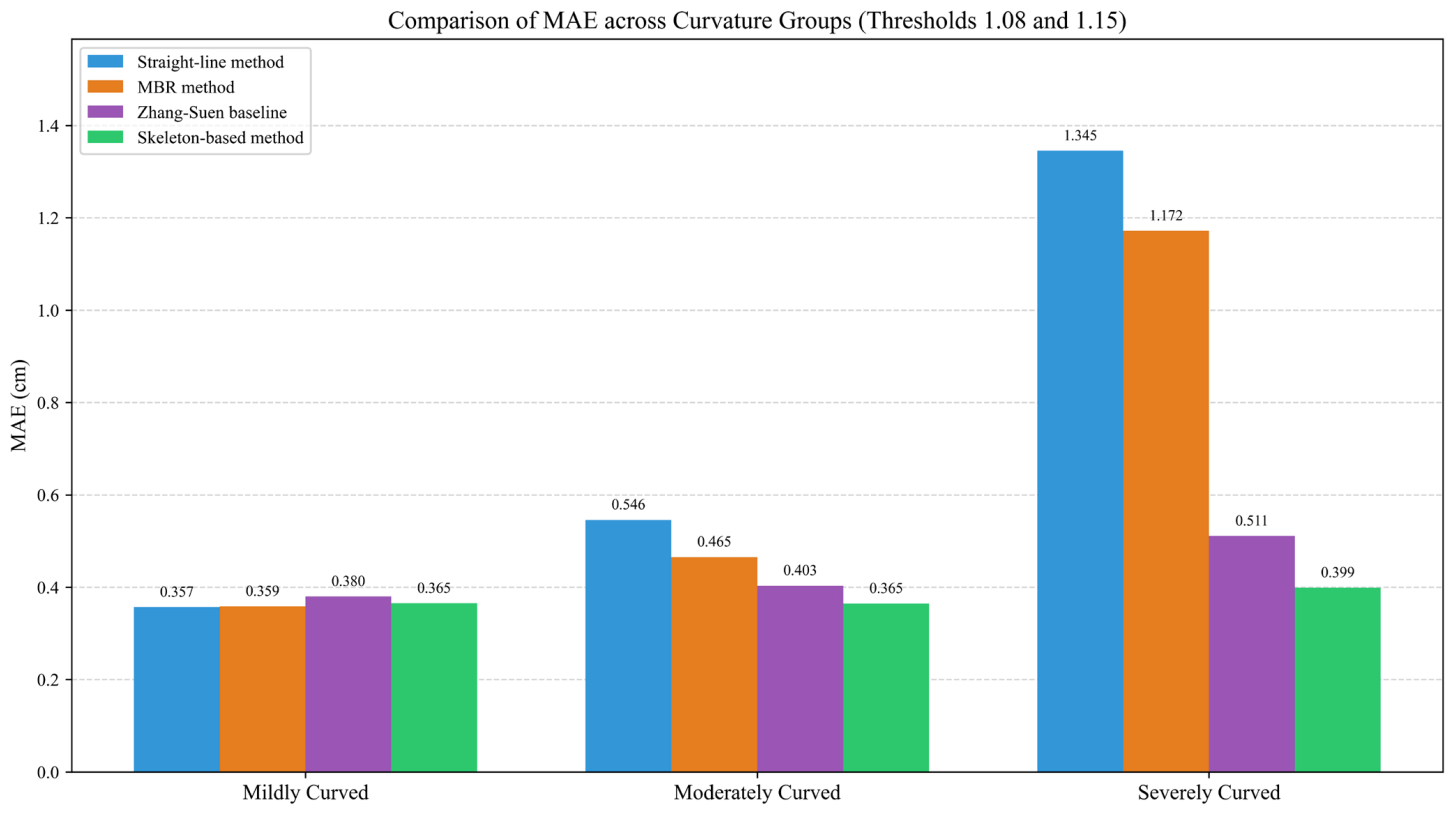
Comparison of MAE among four length measurement methods across different curvature groups.

The comparison of MAE for the four methods across different curvature groups is shown in Figure 7. In the mildly curved group, the MAE values of all four methods were relatively similar. As curvature increased, the error of the straight-line and MBR methods increased markedly. The Zhang–Suen baseline produced lower errors than the two rigid methods but higher errors than the skeleton-based method in the moderately and severely curved groups. In the severely curved group, the skeleton-based method achieved an MAE of 0.399 cm, representing reductions of 70.3%, 66.0%, and 21.9% relative to the straight-line method, MBR method, and Zhang–Suen baseline, respectively.

Table 2 further summarizes the MAE, MAPE, and RMSE of the four methods across different curvature groups. The MAPE of the skeleton-based method remained below 2.76% across all curvature groups, and the RMSE was consistently below 0.49 cm. In the severely curved group, it reduced MAE, MAPE, and RMSE by 21.9%, 21.7%, and 23.1%, respectively, relative to the Zhang–Suen baseline.

### 3.4. Comparison of Body Mass Prediction Performance Between Side-View and Top-View Perspectives

During the construction of the single-view body mass prediction dataset, 18 individuals with incomplete length measurement data were further excluded from the aforementioned 300 samples due to the inability to clearly determine their viewpoint or the absence of valid weighing data. After exclusion, 282 valid samples were obtained, including 170 side-view samples and 112 top-view samples. The mean body mass values of the two groups were 16.23 g and 16.01 g, respectively, and the standard deviations were 4.77 g and 5.02 g, respectively. An independent-samples *t*-test did not detect a statistically significant difference between the two groups (*p* = 0.714).

To systematically compare the influence of different imaging viewpoints on body mass prediction accuracy, we constructed five regression models for each dataset: PLR, MLR, SVR, RF, and XGBoost. Approximately 10% of the samples from each dataset were randomly selected as independent test sets (17 samples for side-view and 12 samples for top-view), while the remaining samples were used for repeated five-fold cross-validation.

Table 3 summarizes the repeated five-fold cross-validation results. Among the five regression models, RF yielded the lowest mean prediction errors under both viewpoints, whereas XGBoost provided competitive but consistently higher errors than RF.

The evaluation results on the independent test sets are shown in Table 4. RF yielded the lowest numerical errors under both viewpoints. For the side-view RF model, the MAPE was 5.73% and the MAE was 0.82 g. For the top-view RF model, the MAPE was 5.82% and the MAE was 1.04 g. The bootstrap 95% confidence intervals for RF MAPE were 3.826–7.727% for the side-view model and 4.372–7.421% for the top-view model. None of the paired comparisons between RF and the other four models reached statistical significance after Holm correction (side-view adjusted *p* = 0.286–0.361; top-view adjusted *p* = 0.256–0.798). Based on the cross-validation and independent-test results, RF was selected as the representative model for subsequent SHAP analysis.

**Figure 8:**
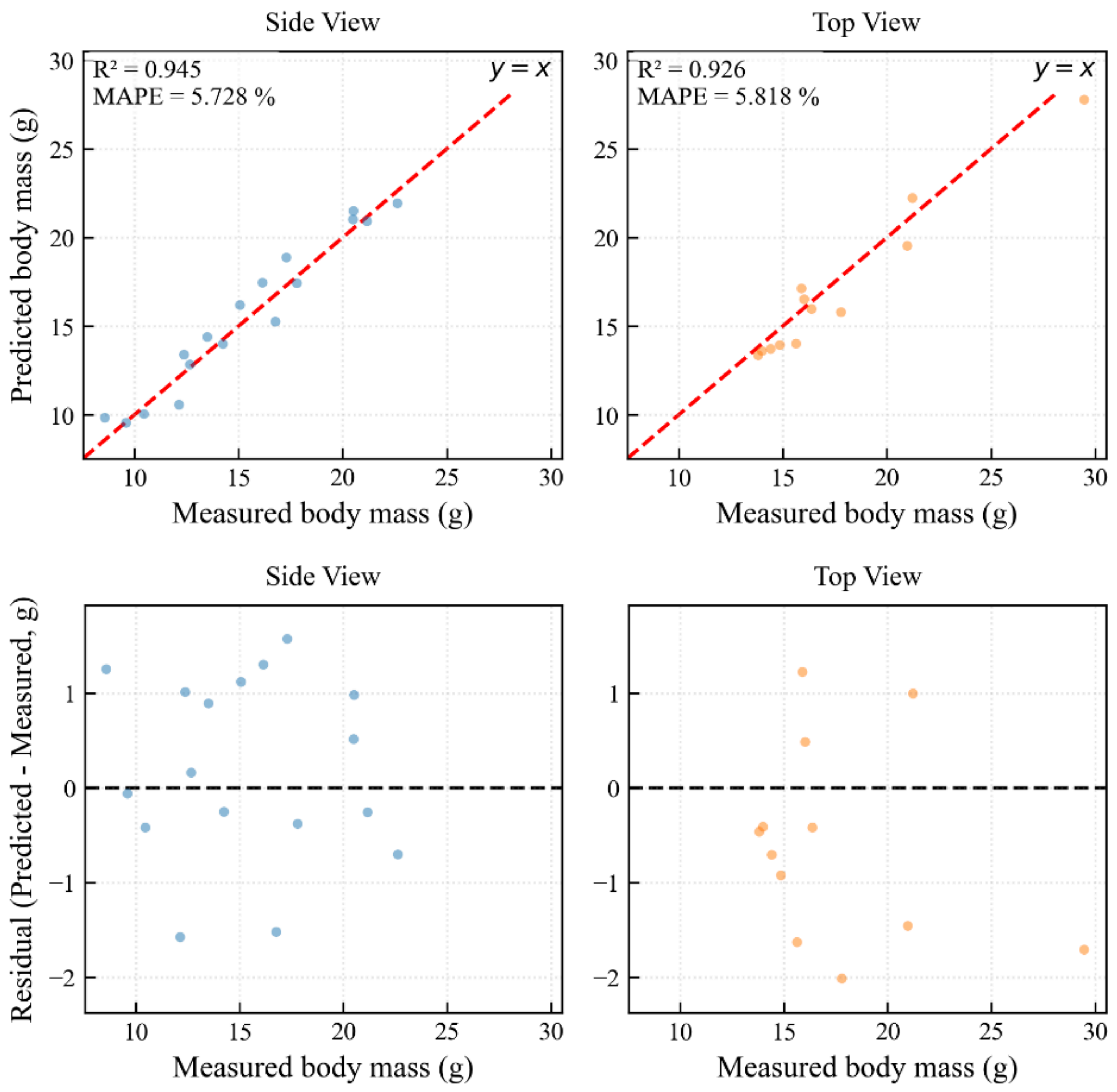
Prediction performance of side-view and top-view RF models. Blue and orange points represent side-view and top-view samples, respectively. The red dashed line denotes y=x in the prediction plots, and the black dashed line denotes zero residual i Confirmed. The revised placement of Figure 9 is acceptable. n the residual plots.

Figure 8 shows the predicted versus measured body mass values and the residual distributions of the RF models on the independent test sets. Under both viewpoints, most observations were distributed near the y=x line, and the residuals were centered near zero. Several larger deviations occurred at higher body masses, indicating that model performance in the upper body-mass range requires further evaluation using additional independent samples.

**Figure 9:**
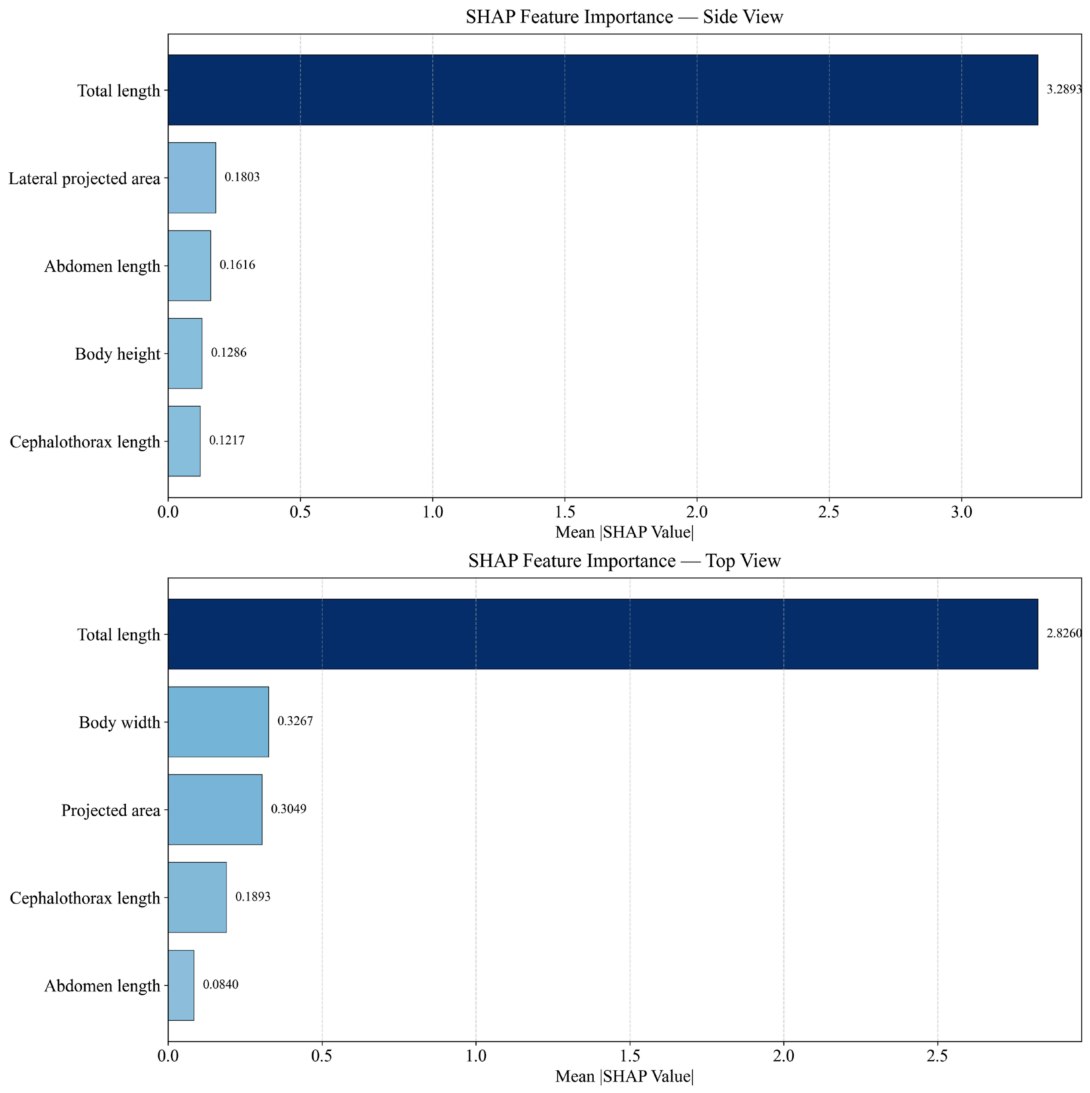
SHAP analysis of side-view and top-view RF models.

To further examine feature use under the two viewpoints, we performed SHAP analysis on the fitted side-view and top-view RF models. Figure 9 presents the mean absolute SHAP value of each feature (g), representing the average magnitude of its contribution to the model output. Within the fitted RF models, total length had the largest mean absolute SHAP value (3.29 g for side-view and 2.83 g for top-view data), whereas the remaining features had smaller values.

### 3.5. Performance of the Unified Model for Mixed Postures

We trained an RF model on the mixed dataset using the unified features (total length, cephalothorax length, abdomen length, maximum transverse dimension, and projected area). For paired comparisons, the unified model and each viewpoint-specific model were evaluated on identical samples within the corresponding viewpoint-specific test subset: 17 side-view samples for the comparison with the side-view-specific model and 12 top-view samples for the comparison with the top-view-specific model. These 29 samples were the same independent test samples used for the single-view models in Section 3.4. The unified model achieved an overall MAPE of 5.54%, an MAE of 0.83 g, and an RMSE of 1.04 g. The overall and view-specific results of the unified model, together with the paired comparisons, are presented in Table 5.

On the side-view subset, the unified and side-view-specific models yielded MAPEs of 6.08% and 5.73%, respectively. The paired MAPE difference was 0.35 percentage points (95% bootstrap confidence interval: −0.74 to 1.38) and was not statistically significant after Holm correction (adjusted *p* = 0.603). On the top-view subset, the corresponding MAPE values were 4.79% and 5.82%, with a paired difference of −1.03 percentage points (95% bootstrap confidence interval: −2.38 to 0.28; adjusted *p* = 0.603). Thus, neither comparison reached statistical significance.

In the sample-size-matched ablation experiment, the unified model yielded mean MAPEs of 6.24 ± 0.53% and 6.26 ± 0.89% for the side-view and top-view subsets, respectively, compared with 5.73% and 5.82% for the corresponding viewpoint-specific models. The matched unified model achieved a lower MAPE in 17.5% of the side-view repetitions and 30.5% of the top-view repetitions. Therefore, the numerical advantage observed for the full unified model on the top-view subset did not persist consistently after controlling for training sample size. The ablation results are summarized in Table 6.

**Figure 10:**
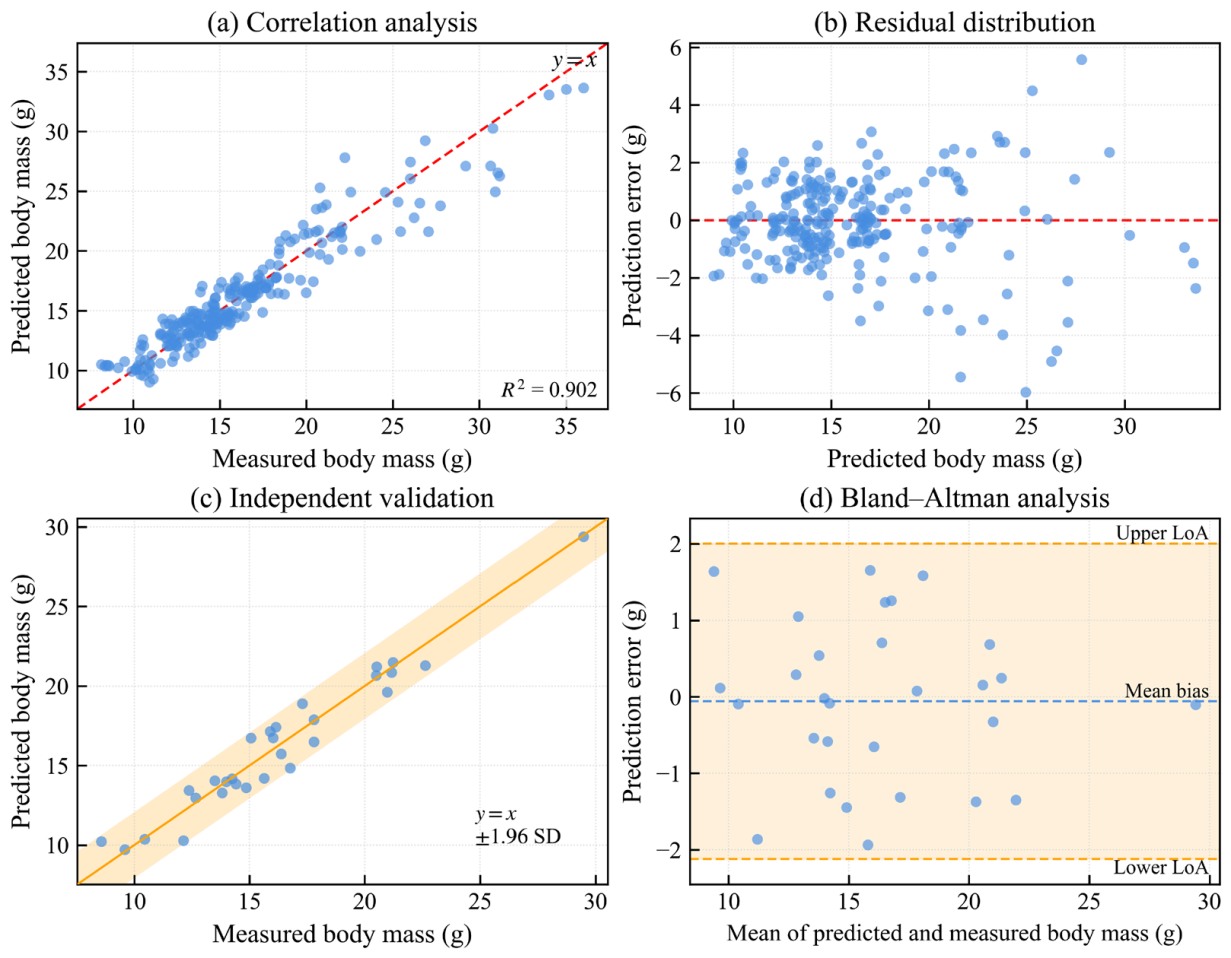
Multidimensional validation of the mixed-posture unified model. (**a**) Cross-validated predictions versus measured body mass. (**b**) Prediction errors, calculated as predicted minus measured body mass, versus predicted body mass. (**c**) Predictions on the independent test set with the y=x reference line and a ±1.96 SD error band. (**d**) Bland–Altman agreement analysis, with differences calculated as predicted minus measured body mass.

Figure 10 summarizes the predictive agreement and error characteristics of the mixed-posture unified model. The model showed close overall agreement between predicted and measured values during cross-validation, with an $R^{2}$ value of 0.902. Residuals were centered near zero over most of the body-mass range, although several larger negative residuals occurred at higher estimated masses.

On the independent test set, the mean prediction error, calculated as predicted minus measured body mass, was −0.06 g, and all samples fell within the calculated 95% limits of agreement. These results indicate limited systematic bias in the present test set, while evaluation of heavier specimens requires a larger independent sample.

These results indicate that the unified feature framework enables a single RF model to predict body mass for both side-view and top-view samples without prior viewpoint identification. On the present test subsets, the paired analyses did not detect statistically significant differences between the unified and corresponding viewpoint-specific models.

## 4. Discussion

This study proposes an automated prawn phenotypic measurement and body mass prediction framework that integrates skeleton-based nonlinear length measurement with multi-view comparative analysis. The framework covers curved total length measurement, comparison of body mass prediction under two viewpoints, and unified prediction for mixed postures. Under controlled imaging conditions, the proposed framework achieved low length-measurement errors and consistent body mass prediction performance across repeated validation.

The curvature index enabled the performance of the four length measurement methods to be compared across different curvature levels. As curvature increased, the errors of the straight-line and MBR methods increased markedly, whereas the skeleton-based method maintained relatively stable errors. Because the extracted skeleton path follows the projected body centerline, it reduces the length underestimation associated with rigid straight-line and bounding-rectangle measurements under curved postures. In the severely curved group, the skeleton-based method achieved an MAE of 0.399 cm relative to the manual two-segment reference and produced lower errors than the three comparison methods. This provides a methodological reference for extending skeleton-based approaches to other flexible aquatic organisms for total length measurement.

Previous studies have applied binary-image thinning to principal-skeleton extraction in prawns [16], combined instance segmentation with skeletonization or centerline prediction [15,17], and optimized centerline extraction for overlapping fish images [18]. These studies demonstrate that skeleton- and centerline-based measurement pipelines can be adapted to different aquatic organisms and imaging conditions. Building on this foundation, the present study evaluates a regional-mask and branch-pruning strategy across predefined curvature groups and provides a controlled comparison with an unpruned Zhang–Suen baseline. To enable a direct method-level comparison, an unpruned Zhang–Suen implementation was included as the skeletonization baseline. The baseline extracted the skeleton path directly from the full prawn mask without branch pruning, whereas the skeleton-based method used separate cephalothorax and abdomen masks and removed short branches before path extraction. The lower errors of the skeleton-based method in the moderately and severely curved groups indicate that regional mask extraction and branch pruning improved centerline localization and reduced interference from appendage-derived branches. These results demonstrate the value of regional mask extraction and branch pruning for robust skeleton-based length measurement of curved prawns.

Regarding viewpoint selection in body mass prediction, the side-view and top-view RF models yielded numerically comparable errors on their respective independent test subsets, with MAPE values of 5.73% and 5.82%, respectively. Because the two subsets comprised different individuals and contained only 17 and 12 samples, respectively, this numerical proximity should not be interpreted as formal evidence of equivalence between viewpoints. Among the five regression models, RF yielded the lowest numerical errors in both repeated cross-validation and independent testing. None of its pairwise comparisons with PLR, MLR, SVR, or XGBoost reached statistical significance after Holm correction. This result is likely related to the limited statistical power of the small independent test subsets. Consequently, the numerical advantage of RF should be interpreted cautiously and requires confirmation using a larger independent dataset.

SHAP analysis showed that total length made the largest contribution under both viewpoints, consistent with the established allometric relationship between body length and body mass in aquatic organisms. In the present study, this agreement provides an internal consistency check on model behavior rather than evidence of a new morphological relationship. SHAP was used for post-hoc interpretation of feature use and not for model selection or architectural justification. The remaining morphological variables contributed to the predictions to different extents, providing a descriptive comparison of feature use between the two imaging perspectives. Independent of the SHAP ranking, the prediction results yielded comparable numerical error levels under the two viewpoints, supporting the practical feasibility of both imaging configurations within the present evaluation.

On the present test subsets, paired comparisons did not detect statistically significant differences between the unified and corresponding viewpoint-specific RF models. The unified model trained on the full pooled dataset yielded a slightly higher MAPE for side-view samples and a lower MAPE for top-view samples. However, the numerical reduction observed for the top-view subset was not consistently reproduced in the sample-size-matched experiment. Accordingly, the principal practical value of the unified framework lies in providing a common prediction model for both viewpoints and simplifying deployment in applications involving mixed prawn postures.

This study has several limitations. First, the independent test set comprised 29 samples. To reduce dependence on a single data split, model stability was additionally evaluated using five-fold cross-validation repeated 10 times, and uncertainty in the independent-test metrics was quantified using bootstrap confidence intervals. Therefore, the independent test set provides an initial out-of-sample evaluation rather than evidence of broad generalization. Future work will collect larger independent datasets covering a wider body-mass range to further validate the models. Second, after stratification by curvature index, the severely curved group (CI > 1.15) contains only 40 individuals, accounting for 13.3% of all valid samples. Although the skeleton-based method yielded lower numerical errors than the three comparison methods in this group, the relatively small sample size may still affect the statistical robustness of this conclusion. Future studies should increase the number of severely curved samples during data acquisition to further strengthen evidence supporting the performance advantage of the skeleton-based method under extreme curvature conditions. Third, all specimens were obtained from one production source and imaged using a single acquisition device under a standardized protocol. These conditions enabled consistent comparison among methods but did not encompass variability across production sources and imaging systems. Further validation using independent datasets acquired with different imaging systems and from additional production sources is warranted to assess the broader applicability of the framework. Fourth, the reference total length was obtained through manual two-segment measurements along the curved prawn body. Although all measurements were performed by the same operator using a standardized procedure, some measurement uncertainty may remain. Future studies may employ three-dimensional scanning or dual-plane imaging to provide an independent reference for length evaluation.

The proposed framework requires sufficiently clear body contours for reliable segmentation, skeleton extraction, and feature computation. In addition, body mass measurements of live prawns may be influenced by gastrointestinal contents and surface-adhering water. Future studies may incorporate multi-view or three-dimensional imaging to obtain volumetric features and further improve body mass prediction.

## 5. Conclusions

This study developed an automated framework for prawn phenotypic measurement and body mass prediction by integrating skeleton-based nonlinear length measurement with multi-view comparative analysis. The framework addresses errors caused by natural body curvature in rigid length measurement and accommodates side-view and top-view postures in image-based body mass prediction. Within the two-dimensional imaging setup, the skeleton-based method combined Zhang–Suen thinning, branch pruning, graph-based shortest-path search, and curvature-based stratification and produced lower measurement errors than the straight-line, MBR, and Zhang–Suen baseline methods, particularly for moderately and severely curved samples.

On this basis, we constructed a multidimensional morphological feature set and compared feature contributions to body mass prediction under the two viewpoints. We further developed a unified mixed-posture model using consistent feature definitions.

For severely curved prawns (CI > 1.15), the skeleton-based method achieved an MAE of 0.399 cm against the manual two-segment reference, representing reductions of 70.3%, 66.0%, and 21.9% relative to the straight-line method, MBR method, and Zhang–Suen baseline, respectively. Among the five regression models evaluated, RF yielded the lowest numerical errors, with MAPE values below 5.9% under both viewpoints, although its paired comparisons with the other models were not statistically significant after Holm correction.

On the present dataset, paired comparisons did not detect statistically significant differences between the mixed-posture unified RF model and the corresponding viewpoint-specific models. The unified feature definition therefore provides a feasible basis for predicting body mass from both side-view and top-view samples using a single model and may simplify deployment in applications involving mixed prawn postures.

## Figures and Tables

**Table 1:** Performance of YOLO11n-seg instance segmentation model.

Type	Dice	ASD /Pixel	HD95 /Pixel	Mask mAP@50:95
Head	0.931	3.212	10.755	—
Abdomen	0.950	1.935	6.996	—
Mean	0.940	2.573	8.876	0.840

**Table 2:** Comparison of four length measurement methods across different curvature groups.

Curvature Group	Metrics	Straight-Line Method	MBR Method	Zhang–Suen Baseline	Skeleton-Based Method
Mildly curved	MAE/cm	0.357	0.359	0.380	0.365
	MAPE/%	2.520	2.550	2.660	2.594
	RMSE/cm	0.469	0.474	0.450	0.467
Moderately curved	MAE/cm	0.546	0.465	0.403	0.365
	MAPE/%	3.702	3.168	2.833	2.559
	RMSE/cm	0.680	0.585	0.516	0.462
Severely curved	MAE/cm	1.345	1.172	0.511	0.399
	MAPE/%	9.103	7.912	3.519	2.755
	RMSE/cm	1.477	1.321	0.628	0.483

**Table 3:** Performance of the body mass prediction models obtained using repeated five-fold cross-validation.

View	Metrics	PLR	MLR	SVR	RF	XGBoost
Side view	MAPE/%	8.007 ± 0.973	9.098 ± 1.543	9.663 ± 1.842	7.249 ± 0.878	7.972 ± 1.241
	MAE/g	1.327 ± 0.210	1.437 ± 0.189	1.604 ± 0.310	1.222 ± 0.176	1.342 ± 0.232
	RMSE/g	1.852 ± 0.327	2.024 ± 0.335	2.560 ± 0.615	1.626 ± 0.267	1.824 ± 0.367
Top view	MAPE/%	7.120 ± 1.598	8.149 ± 1.631	9.204 ± 2.345	7.114 ± 1.366	7.856 ± 1.347
	MAE/g	1.156 ± 0.302	1.228 ± 0.239	1.683 ± 0.693	1.088 ± 0.222	1.217 ± 0.216
	RMSE/g	1.682 ± 0.573	1.578 ± 0.355	3.081 ± 1.763	1.385 ± 0.292	1.577 ± 0.324

Values are presented as the mean ± standard deviation across 50 validation folds obtained from five-fold cross-validation repeated 10 times.

**Table 4:** Performance of the body mass prediction models on the independent test sets.

View	Metrics	PLR	MLR	SVR	RF	XGBoost
Side view	MAPE/%	9.302 (6.100–13.131)	11.276 (5.952–18.602)	10.485 (5.903–16.209)	5.728 (3.826–7.727)	7.085 (4.783–9.736)
	MAE/g	1.216 (0.898–1.537)	1.472 (0.907–2.149)	1.341 (0.878–1.852)	0.822 (0.584–1.057)	1.012 (0.694–1.368)
	RMSE/g	1.396 (1.092–1.669)	1.978 (1.159–2.783)	1.681 (1.097–2.198)	0.963 (0.729–1.163)	1.240 (0.836–1.599)
Top view	MAPE/%	7.260 (5.075–9.709)	7.767 (4.643–11.252)	9.123 (6.065–13.553)	5.818 (4.372–7.421)	7.106 (4.640–9.569)
	MAE/g	1.383 (0.816–2.181)	1.427 (0.772–2.211)	1.821 (0.987–3.179)	1.036 (0.742–1.347)	1.200 (0.790–1.622)
	RMSE/g	1.857 (0.917–2.823)	1.907 (1.098–2.585)	2.734 (1.105–4.376)	1.168 (0.852–1.456)	1.396 (0.977–1.754)

Values are point estimates with percentile-based bootstrap 95% confidence intervals in parentheses.

**Table 5:** Performance of the unified RF model and paired comparisons with the viewpoint-specific RF models on the independent test set.

Test Set	Unified RF			Viewpoint-Specific RF	Paired Comparison			
	MAPE/%	MAE/g	RMSE/g	MAPE/%	ΔMAPE/pp	95% Bootstrap Confidence Interval of ΔMAPE/pp	*W*	Adjusted *p*
Side view	6.080	0.864	1.102	5.728	0.352	[−0.741, 1.378]	64	0.603
Top view	4.786	0.790	0.933	5.818	−1.033	[−2.376, 0.282]	25	0.603
Overall	5.544	0.834	1.036	—	—	—	—	—

ΔMAPE was calculated as the unified RF MAPE minus the viewpoint-specific RF MAPE. Positive values indicate a higher error for the unified model. Confidence intervals were estimated using 2000 paired bootstrap resamples. *W* denotes the Wilcoxon signed-rank statistic. *p*-values were adjusted using the Holm method. pp, percentage points.

**Table 6:** Results of the repeated sample-size-matched ablation experiment.

Test Set	Matched Unified RF MAPE/%	Specific RF MAPE/%	Mean ΔMAPE/pp	2.5th–97.5th Percentiles of ΔMAPE/pp	Runs with Lower Unified MAPE/%
Side view (matched *n* = 153)	6.240 ± 0.533	5.728	0.513	[−0.383, 1.767]	17.5
Top view (matched *n* = 100)	6.255 ± 0.893	5.818	0.437	[−1.151, 2.273]	30.5

Results for the matched unified model are presented as the mean ± SD across 200 repetitions. The pooled training set was sampled without replacement and stratified by viewpoint. ΔMAPE was calculated as the matched unified-model MAPE minus the viewpoint-specific-model MAPE. The percentile interval represents the empirical distribution of the repeated downsampling results and is not a confidence interval based on independent test samples. pp, percentage points.

## Data Availability

All data that directly support the findings of this study are fully presented within the article (Table 1, Table 2, Table 3, Table 4, Table 5 and Table 6 and Figure 1, Figure 2, Figure 3, Figure 4, Figure 5, Figure 6, Figure 7, Figure 8, Figure 9 and Figure 10). Additional details, such as individual measurement records, are available from the corresponding author upon reasonable request.
